# Re-Whole Brain Radiotherapy for Brain Metastases: How to Evaluate an Outcome of Repeat Treatment When Its First Use Is Debatable

**DOI:** 10.3390/cancers15061790

**Published:** 2023-03-16

**Authors:** Lucyna Kępka

**Affiliations:** Department of Radiotherapy, Military Institute of Medicine—National Research Institute, 04-141 Warsaw, Poland; lkepka@wim.mil.pl

Recently, a review on a pertinent issue of repeat whole-brain radiotherapy (re-WBRT) for recurring brain metastases was published [1]. Based on 19 retrospective studies on re-WBRT, it was concluded that re-WBRT may be an option for symptomatic recurrent brain metastases; however, asymptomatic patients should not undergo re-WBRT due to insufficient data on late toxicity. It was also noted that re-WBRT has limited acute toxicity and improves symptoms in some proportion of patients. Of note, all except 2 reviewed studies were published more than 5 years ago, including 6 studies from the 20th century. This may reflect a changing attitude toward WBRT challenging its use based on its neurotoxicity and lack of improvement in overall survival (OS) compared with local treatment (stereotactic radiosurgery [SRS] or surgery) with omission of WBRT [2,3,4]. Additionally, other treatment methods directed at multiple brain metastases, such as SRS of multiple lesions, and innovations in systemic treatment, such as targeted therapy and immunotherapy, are in increased use. Thus, one may argue that re-WBRT is currently seldom prescribed. However, some symptomatic patients are still not suitable for multiple lesions SRS due to the extent and type of dissemination in the brain and for whom there are no systemic therapy options. In such a clinical scenario, the conclusions of this review regarding a possible administration of re-WBRT are of value.

For symptomatic patients with multiple brain metastases not suitable for SRS, WBRT is recommended at baseline because data show it can bring a symptomatic improvement [5]. However, there is no evidence for its benefit over best supportive care (BSC) alone with the administration of steroids. The Quality of Life after Treatment for Brain Metastases (QUARTZ) study randomized 538 patients with brain metastases from NSCLC, ineligible for SRS or surgery, to BSC, including dexamethasone plus WBRT or BSC (including dexamethasone) alone. There was no difference in OS, quality-adjusted life year, and, notably, in the steroid use between the arms of the study [6]. In another prospective study, the value of WBRT for symptom management in poor performance status (PS) patients (RTOG RPA class 3) was evaluated. A total of 91 patients received WBRT (20 Gy in 5 fractions), and were asked to complete a questionnaire about their symptoms before and one month after WBRT. A total of 43 (47%) patients completed both symptom checklists. The remaining died within one month or their condition deteriorated to the point that they were unable to complete a symptom checklist with 17 items scored from 0 to 3. The mean scores on the symptom checklist were 18.21 and 21.09 at baseline and 1 month after WBRT, respectively (*p* = 0.02). A higher score meant a greater symptom intensity, indicating a worsening of patients’ symptoms in the subgroup of one-month survivors [7]. Thus, little, if any, clinical benefit of WBRT in patients with unfavorable prognosis was demonstrated in the prospective studies [6,7]. Physicians have a tendency to overestimate the clinical benefit of WBRT. In one study, physicians referring patients with brain metastases for consideration of WBRT completed surveys about their expectations on the effect of WBRT for respective patients. Of the referring physicians, ≥95% thought that WBRT would stabilize neurologic symptoms, improve PS, enhance quality of life (OoL), and allow for the tapering of steroids. They estimated patient OS would be a median of 6.0 months; however, the actual median was 2.5 months. Steroid tapering did not occur in the majority of patients after WBRT. Physicians tend to estimate the benefit of proposed strategies much more positively than what is realistic, potentially resulting in aggressive therapies without benefit [8]. This risk may be even greater for re-WBRT, which is directed at patients with a generally poorer prognosis than those before a first WBRT.

The issue of toxicity in re-WBRT is crucial. I agree that we have no data on late toxicity in these patients due to their very short survival. We may disregard this kind of toxicity, at some point, taking into account that we only deal with patients’ symptoms in the short term; however, the issue of acute toxicity cannot be neglected. In palliative care, we are very careful about patients’ QoL. The studies included in the review concluded that acute toxicity was acceptable; however, only side effects of WBRT managed by steroids, such as headache, nausea, and deterioration of neurological status, were evaluated. Other symptoms, often reported by patients after WBRT, such as fatigue, appetite loss, daily somnolence, and cognitive disturbances, were not considered in most of the included series. Such symptoms may severely impair QoL of patients, and, in the context of limited survival, patients do not recover from this acute toxicity before their death. Measurement of QoL and the toxicity after WBRT for brain metastases give controversial results [9,10]—these patients may deteriorate not because of the side effects of WBRT, but despite WBRT because of intracranial or extracranial progression. Recognizing all of these limitations, we should be aware that when making a decision about re-WBRT that not only late, but also acute toxicity and other side effects related to the impairment of QoL may be underestimated. Therefore, the results of the ongoing randomized study (NCT03288272) comparing re-WBRT in 2 different fractionation schedules (20 Gy in 10 fractions vs. 30 Gy in 15 fractions) with BSC only, with an evaluation of the toxicity as primary end-point (QoL and survival being secondary end-points), are eagerly expected.

Based on the reviewed articles, considering radiobiological data on the risk of the use of high fractional doses, and no evidence of the benefits of dose escalation in WBRT, the authors propose for re-WBRT a fractionation schedule of 20 Gy in 10 fractions or 18 Gy in 5 fractions within the tolerable total biological effective dose for brain (for a sum of both WBRT courses) of ≤150 Gy. The choice of more protracted schedules results from some apprehension with regard to late brain toxicities related to the use of high fractional doses. This is also a common practice in the case of reirradiation of larger volumes in extracranial locations. On the other hand, the short expected survival of most of these patients may induce physicians to shorten the treatment duration in order to spare them some discomfort related to the repeated visits and transport to radiotherapy facilities. Therefore, the 5 fractions schedule, e.g., 18 Gy/5 fractions, may be more convenient than 10 fractions for patients. Recently, it was shown, in a post hoc analysis of NCCTG N107C [Alliance]/CEC.3 prospective trial, that, in 92 patients receiving WBRT after surgical resection of brain metastasis, there was no significant difference in time to cognitive failure, surgical bed control, intracranial brain control, and OS between 49 patients treated with 30 Gy in 10 fractions and 43 treated with 37.5 Gy in 15 fractions. Moreover, the adverse effects of WBRT were significantly higher with a more protracted radiation schedule [11].

Another issue around making decisions about the qualification for re-WBRT more complex is the lack of prognostic indices we have at our disposal for brain metastases patients referred for WBRT at baseline. The scales are derived from a recursive partitioning analysis (RPA) that divided patients into three prognostic groups based on the PS, control of extracranial disease, and age [12]. Next, the prognostic value of a number of brain metastases was confirmed, and a graded prognostic assessment (GPA), with the addition of this new factor, was proposed [13]. However, prognostic factors may vary by diagnosis; thus, the diagnosis-specific GPA for lung, renal, breast, and gastrointestinal cancers was created and validated [14]. Finally, for NSCLC and melanoma, molecular markers were added to develop molecular GPA. The presence of epidermal growth factor receptor and anaplastic lymphoma kinase rearrangement were recognized for lung adenocarcinoma as positive prognostic factors [15,16]. Recently, in brain metastases from NSCLC-adenocarcinoma, PD-L1 status was identified as a significant prognostic factor, and the previously identified molecular factors were reaffirmed [17]. In the case of re-WBRT, we have no distinct prognostic groups. At the relapse of brain metastases, the clinical value of current prognostic scales is most likely limited, though the most powerful prognostic factor, overall PS, may drive clinical decisions. In a series of 205 patients receiving re-WBRT, application of the RPA score was not predictive of OS. However, the main prognostic factors from the RPA score—PS and control of extracranial disease—correlated with OS. Additionally, the interval between courses of WBRT, <9 months, a small cell lung cancer histology significantly correlated with the shorter OS. By assigning a score of 1 to each of these factors, a new prognostic reirradiation (ReRT) score was proposed [18]. In the commented review, a longer interval between two courses of WBRT is also indicated as a possibly positive prognostic factor. The reviewed series had no data on the molecular status of the respective diagnoses. Longer intervals between courses of WBRT may reflect an inherent favorable disease course driven by molecular factors that determine the degree of aggressiveness of brain metastases and the response to therapeutic interventions.

Despite all limitations related to the characteristic of the reviewed studies, existing evidence on the limited value of WBRT in terms of expected symptomatic improvement and lack of effect on the tapering of steroids, together with demonstrated neurotoxicity, I hope that a review by Ono and Nemoto, providing a summary of the existing data on re-WBRT, will serve the readership in the shared decision making with patients when no other treatment method than WBRT is feasible.

When acknowledging the limited evidence, the incorporation of the patient’s values and preferences into the decision about treatment is of crucial importance.
